# Using the Radial-Shear Rolling Method for Casted Zirconium Alloy Ingot Structure Improvement

**DOI:** 10.3390/ma17205078

**Published:** 2024-10-18

**Authors:** Alexandr Arbuz, Fedor Popov, Alexandr Panichkin, Anna Kawałek, Nikita Lutchenko, Kirill Ozhmegov

**Affiliations:** 1 Core Facilities—Office the Provost, AEO Nazarbayev University, 53 Kabanbay Batyr Ave, Astana 010000, Kazakhstan; fedor_popoff@mail.ru (F.P.); nikita.lutchenko@nu.edu.kz (N.L.); 2 Institute of Metallurgy and Ore Benefication JSC, Satbayev University, 29/133 Shevchenko St., Almaty 050010, Kazakhstan; a.panichkin@satbayev.university; 3 Department of Production Management, Faculty of Engineering Production and Materials Technology, Częstochowa University of Technology, ul. J.H. Dąbrowskiego 69, 42-201 Częstochowa, Poland; anna.kawalek@pcz.pl (A.K.); kvozhmegov@wp.pl (K.O.)

**Keywords:** radial-shear rolling, microstructure, plastic deformation, ingot, cast structure, grain refinement

## Abstract

In developing materials for the nuclear industry, it is crucial to enhance both alloy composition and processing methods. This study focuses on investigations of applying radial-shear rolling (RSR) to a Zr-1%Nb alloy ingot, aiming to refine its microstructure and improve its properties for nuclear applications. This method, with complex vortex metal flow inside of a casted workpiece, has not been previously tested for processing zirconium ingots, so experimental verification of its applicability is of scientific interest. The 30 mm diameter ingot, produced by vacuum induction melting, was initially rolled to 20 mm at 800 °C to eliminate defects and refine the cast structure. A second rolling stage reduced the diameter to 13 mm at 530 °C, resulting in an ultrafine-grained structure. The RSR method effectively combines structural refinement and defect healing within fewer cycles, making it suitable for producing components for nuclear reactors. This approach demonstrates a potential reduction in traditional processing steps, providing a more efficient route for preparing high-quality materials for nuclear applications.

## 1. Introduction

Zirconium alloys play a key role in the nuclear power industry due to their unique physicochemical and operational characteristics [1,2,3]. These alloys are widely used for the manufacture of structural elements of fuel assemblies of nuclear reactors due to their high corrosion resistance, low neutron capture cross-section (0.18 barns for zirconium compared to 2.2 barns for stainless steel) [4], and high resistance to radiation damage [5,6]. The alloy of the Zr-1%Nb alloy stands out among other zirconium alloys due to its improved mechanical properties, including yield strength of about 450 MPa and ultimate tensile strength of 650 MPa [7], and high resistance to water corrosion, which makes it especially in demand in various types of reactors, including light-water reactors (LWRs) [8] and boiling water reactor (BWR) [9].

It is also important to note that there is a constant search for alternative materials, such as molybdenum-based alloys with a eutectic microstructure that have excellent corrosion resistance. Thus, in studies of the phase field of the evolution of the microstructure of the eutectic transformation and the four-phase reaction in the Mo-Si-Ti system [10], important mechanisms of microstructure formation were identified that affect the improvement in the properties of these alloys. In addition, the study of the oxidation and creep characteristics of new Mo-Si-Ti alloys [11] showed their high potential for use in high-temperature and aggressive environments that are extremely high at nuclear power plants [1]. The ingot processing method we propose can also be very useful for these alloys. However, since zirconium alloys remain dominant in the nuclear industry, it was decided to begin the study with zirconium alloys.

The achievement of high-performance characteristics of zirconium alloys depends not only on their chemical composition but also on the method of their processing [12,13]. In particular, the microstructure formed during the production process plays a key role in ensuring strength, plasticity, and resistance to various types of damage [14]. Therefore, the choice and development of effective methods for processing zirconium alloys are becoming critically important to ensure the reliability and durability of the material, especially in the operating conditions of nuclear reactors [15,16].

**Figure 1:**
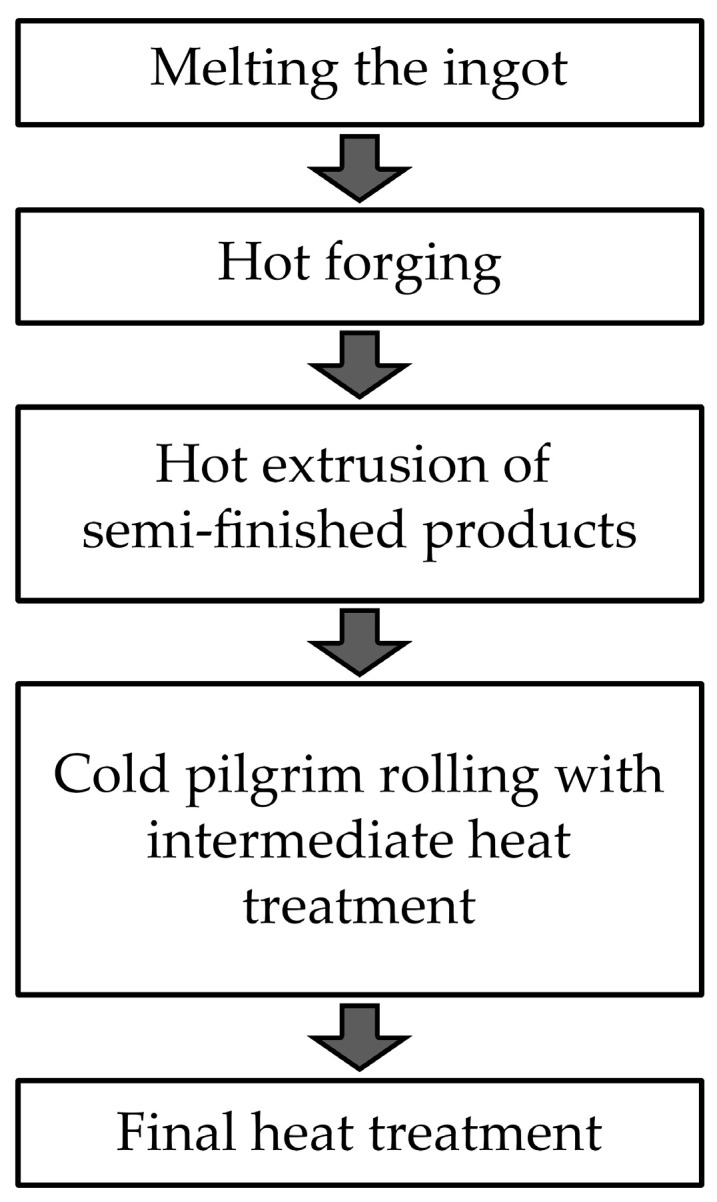
The technology operations of manufacturing tubes and rods from the Zr-1%Nb alloy.

The technology of manufacturing tubes and rods from the Zr-1%Nb alloy includes the following operations (Figure 1). During the hot forging process, the cast structure is fragmented, and the cross-section of the forging is reduced. The next stage is hot extrusion, during which there is a significant change in the cross-section. The next stage is cold rolling with intermediate and final annealing, during which a specific structure and the properties of the finished products are formed.

Conventional methods of thermomechanical processing of zirconium alloys, such as hot rolling and forging, are widely used in industry to improve the microstructure and, consequently, the performance characteristics of the material [17,18,19,20]. These processes are aimed at reducing the grain size of the cast structure, which leads to increased strength, ductility, and resistance to cracking of the material. Hot deformation treatment, for example, is carried out at a temperature above the recrystallization temperature of the material, typically at approximately 800–900 °C [21], which allows for reducing deformation hardening and obtaining a finer and more homogeneous structure [22]. Forging, in turn, makes it possible to improve the density of the material, reducing porosity by up to 70% [23], and eliminates casting pores, which also contributes to improving mechanical properties [24].

However, traditional thermomechanical processing methods have some limitations. They do not ensure grain refinement to a level of less than 1 micron and are characterized by significant metal losses into chips (up to 15% of the initial mass) [25] and a long manufacturing cycle of finished products. Thermomechanical processing by traditional methods does not always allow for controlling the microstructure at the submicron level. For example, studies show that during hot rolling and forging, a significant proportion of the residual cast structure is often preserved, which may include large grains (up to 20 microns) and agglomerates of inclusions of the second phase [26,27]. For the refinement, it is necessary to carry out additional deformation and thermal treatments [28].

In this regard, in recent years, there has been a growing interest in the development and application of new methods of severe plastic deformation (SPD), which make it possible to significantly grind grains and ensure a more uniform structure and properties during ingot shaping [29,30,31]. These methods open up new possibilities for improving the mechanical properties of zirconium alloys, as well as their performance characteristics under conditions of exposure to an aggressive environment (steam, high temperatures, and fluence) [32].

The application of SPD methods to metal ingots is of particular interest since research in this direction is not actively conducted. Studies show that the use of methods of intensive plastic deformation (SPD), such as equal-channel angular pressing (ECAP), high-pressure torsion (HPT), and accumulative rolling bonding (ARB), can significantly improve the properties of cast metals due to intensive grain refinement and elimination of casting defects [33,34,35]. For example, ECAP was successfully used for processing cast aluminum alloys, which led to a decrease in the average grain size from 100–150 microns to 0.5–1 microns, while increasing strength by 50–70% and improving ductility by 20–30% [36]. When processing cast copper and titanium–aluminum alloys by the HPT method, it was possible to achieve an ultrafine-grained structure with a grain size of less than 100 nm, which led to an increase in hardness by 80–100% and a significant decrease in the coefficient of friction under wear conditions [37]. ARB rolling demonstrates efficiency in the processing of cast aluminum and magnesium alloys, where the grain size was reduced from 50–200 microns to 200–400 nm, which contributed to an increase in strength by 30–40% and an improvement in fatigue resistance by 40–60% [38].

The listed SPD methods have several significant disadvantages. Firstly, they are limited by the size of the workpieces to be processed: ECAP and HPT are suitable only for small samples (up to 30 mm in diameter) [39], which makes it difficult to use them for large parts. Secondly, many repetitions of the process (often 4–8 passes) [40] are required to achieve the required microstructure, which greatly complicates the process. However, the main limitation is that these repetitions cannot be integrated into continuous and automated production lines. This makes it difficult to scale and implement SPD methods in production, where high productivity and process continuity are required [34]. These limitations emphasize the need to find new methods that can overcome these problems and provide more efficient and scalable material processing.

To solve these disadvantages, the radial-shear rolling (RSR) method has proven itself well [41,42]. RSR is a plastic deformation process in which the workpiece is subjected to both radial compression and axial shear. During the RSR, the workpiece rotates around its axis and moves along the axis of the rolling mill, which ensures the achievement of very high degrees of deformation (up to 35 mm/mm and above) with a small change in size. Due to the increased angles of inclination of the rolls in the range of 18–21 degrees, the metal in the deformation zone is directed in an axisymmetric vortex flow with a radial gradient. Such a stress–strain state contributes to intense deformation of the outer region of the sample, which leads to the formation of an equiaxed ultrafine-grained structure with grain sizes less than 1 micrometer [43]. In the central zone of the sample, due to the laminar flow of metal along the rolling axis, a rolling texture is formed directed along the rolling direction. Due to this, it is possible to significantly reduce the grain size and improve the properties of the material. The RSR method has obtained good results on titanium [42], stainless steel [41], aluminum [44], magnesium alloys [45], and zirconium alloy Zr-2.5Nb in the annealed state [43].

RSR has several key advantages over traditional SPD methods. Firstly, it is suitable for processing workpieces without length limitations and with much larger sizes (up to 100 mm in diameter) [41,46], which makes it especially effective for industrial scales. Secondly, the RSR process requires fewer passes to achieve the required microstructural changes, resulting in significantly shorter processing times and lower costs [47]. This simplification allows for smoother integration into continuous production lines, making RSR more suitable for large-scale industrial applications compared to ECAP and HPT, which require specialized equipment and have discrete billet sizes. Unlike ECAP and HPT methods, which require complex and expensive equipment and have discrete billet sizes, RSR is highly flexible and can be adapted to existing production lines [46].

Despite the obvious advantages, RSR remains poorly understood in the context of deformation treatment of the cast structure of ingots, especially zirconium-based alloys. The cast structure often has a coarse-grained microstructure and defects, such as pores and segregation of alloying elements that traditional methods may not completely eliminate. Therefore, the study of RSR for such materials is a promising approach that opens up new opportunities for improving the structure and properties of finished products. This is especially important for high-tech industries, such as nuclear power, where the requirements for structural materials are critically important; the material is characterized by high cost and limited quantity [3].

The aim of this work is to study the effect of SPD during radial-shear rolling of a Zr-1%Nb alloy ingot on the cast structure and defect closure, as well as to study to the formation of microstructure and properties depending on thermomechanical processing conditions. When analyzing the results, the article assesses the applicability and effectiveness of the RSR method for rolling ingots, as well as the resulting product for its suitability for further processing.

## 2. Materials and Methods

To carry out this work, an ingot was specially smelted by vacuum induction melting. For testing the technology of zirconium rolling, ingots smelted by this method are quite suitable [48]. The casting mold was made of calcium oxide powder (CaO). CaO powder was rammed into the inner cavity of a quartz tube with a diameter of 100 mm, using a steel cylinder with a diameter of 30 mm to form a casting cavity. After tamping, the cylinder was removed, and the mold was calcined at a temperature of 1140 °C for 3 h. The ingot diameter was determined, taking into account the technical capabilities of the RSR rolling mill, capable of processing workpieces with a maximum diameter of up to 30 mm. A round rolled billet with a diameter of 50 mm and a length of 200 mm made of Zr-1%Nb alloy was used as a charge.

**Figure 2:**
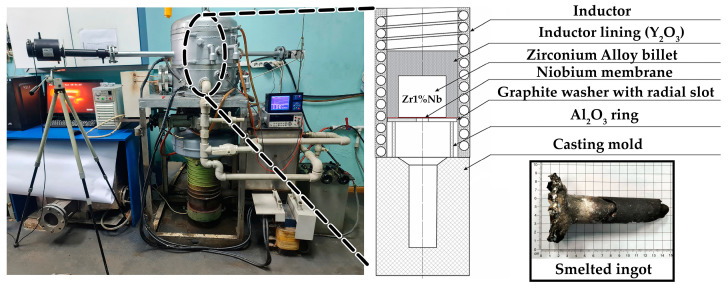
The scheme of the melting vacuum induction furnace.

An induction vacuum furnace was used to melt the zirconium billet. The scheme of the casting module used to drain the zirconium melt through the bottom of the lining is shown in Figure 2. This scheme ensured minimal overheating of the zirconium melt by using an automated drain through a hole at the bottom of the furnace lining.

An alund tube ring with a diameter of 70 mm and a height of 60 mm was installed on the casting mold. A graphite washer with an outer diameter of 100 mm and a central hole with a diameter of 20 mm was placed on top of the ring. To prevent overheating in the induction field, the ring had a radial slot. A membrane made of niobium foil with a thickness of 40 microns was placed on the ring, and then a cylinder made of zirconium was placed over ring. The space between the inductor and the cylinder was filled with pre-calcined calcium oxide powder, which was additionally tamped. The powder was also poured over zirconium, forming a layer 20–25 mm thick.

After melting the zirconium and dissolving the niobium membrane, the melt flowed through the hole, filling the funnel of the mold, and then, under the influence of gravity, the mold itself. The melting and casting of zirconium was carried out after preliminary pumping of air and purging of the furnace chamber with argon. During the melting process, the residual pressure was 10 Pa.

The alloy composition after the remelting is: Zr—97.226%, Nb—1.063%, Fe—0.309%, Cr—0.088%, Ni—0.098%, Cu—0.048%, Nd—0.003%, Al—0.275%, and Si—0.889%. The alloy received some small impurities from the slag; however, for the study of the crushing of the cast dendritic structure, this is not so important.

**Figure 3:**
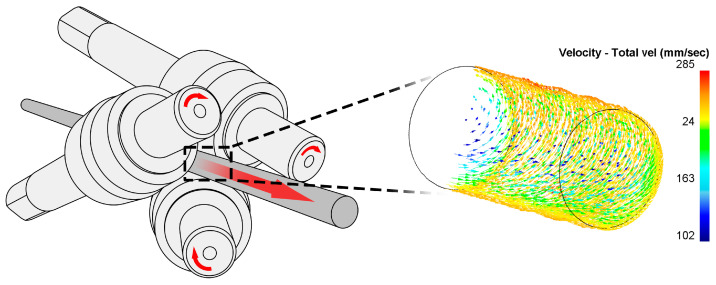
The operation scheme of the rolling mill RSR-10/30 (**left**) and the metal-flow peculiarities inside the workpiece (**right**) [46].

The cast billet was subjected to radial-shear rolling in order to study the cast structure, improve its mechanical properties, and close defects. The experiment was carried out at the radial-shear mill RSR-10/30, located at Karaganda Industrial University (Temirtau, Kazakhstan). The operating scheme of the machine is shown in Figure 3.

The rolling process was carried out in two stages. In the first stage, at a temperature of 800 °C, the diameter of the workpiece was reduced from 30 mm to 20 mm with the maximum possible single reduction of 3–4 mm per step, which should contribute to intensive closure of discontinuities and homogenization of the workpiece. In the second stage, at a reduced temperature of 530 °C, the workpiece was rolled to a diameter of 13 mm in 2 mm increments for intensive structure refinement. The choice of conditions for the second stage is based on the work [49], where an ultrafine-grained structure was obtained under similar conditions.

It was assumed that in the first stage, at elevated temperatures, possible defects would be brewed, transforming a non-plastic brittle cast structure into a suitable one for processing, and in the second stage, this structure would be severely refined due to high deformations and reduced temperatures.

**Figure 4:**
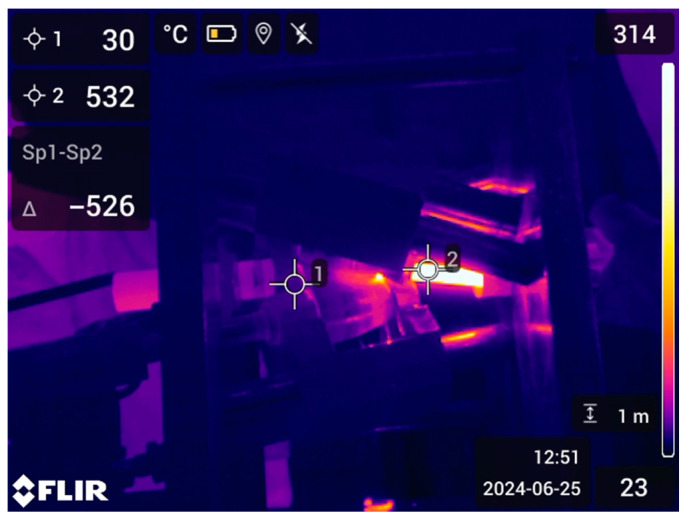
The rolling process at 530 °C (thermal imager control).

**Figure 5:**
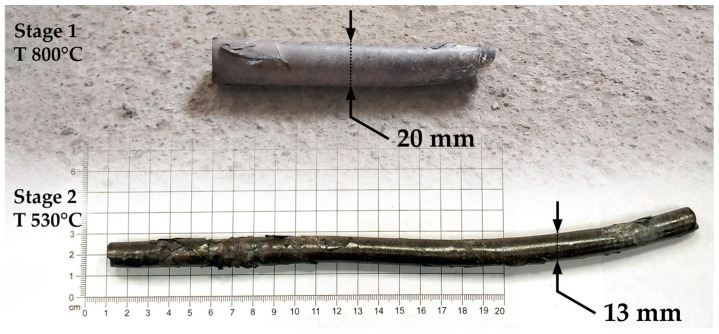
Rolled workpieces: top—up to 20 mm, bottom—13 mm.

For precise temperature control during the rolling process, the Flir T540 (Flir, Wilsonville, OR, USA) thermal camera was used, which recorded temperature changes on the bar’s surface in real time. Figure 4 shows the rolling process controlled using a thermal imager. The processed workpieces are shown in Figure 5.

After rolling, the bar was cut into short, smaller bars for microstructural analysis. Each bar was then halved along its axis using a Brilliant-220 precision cutting machine (QATM, Mammelzen, Germany) with a cutting speed of 10 μm/s and intense water cooling to prevent structural damage from heat and deformation. Two thin plates were cut from each half—one for Transmission Electron Microscopy (TEM, Jeol, Tokyo, Japan) to examine the fine structure of the deformed peripheral zones and the other for Electron Backscatter Diffraction (EBSD) analysis following electrolytic polishing (jet polishing). The longitudinal section was first polished on a Saphire-520 refinement and polishing machine (QATM, Mammelzen, Germany) and subsequently subjected to electrolytic polishing using a LectroPol-5 unit (Struers, Copenhagen, Denmark).

Chemical composition analysis was performed by the wavelength dispersive X-ray Fluorescence (XRF) method on the м (Malvern, Worcestershire, UK).

Electron microscopy techniques were employed to investigate microstructural transformations. The characterization of grain structures, including crystallographic orientation, was performed using Electron Backscatter Diffraction (EBSD) on a Carl Zeiss Crossbeam-540 Field Emission Scanning Electron Microscope (Carl Zeiss AG, Oberkochen, Germany) equipped with an Oxford Instruments NordlysNano EBSD (Abingdon, UK) detector. Data acquisition and analysis were conducted with the free software ATEX v. 4.12 [50], generating maps along a radial line from the center to the periphery at five equidistant points for 20 mm spaced 2 mm apart and for 13 mm spaced 1.625 mm apart.

The microhardness test was conducted on the cross-section plate prepared for EBSD using a Shimadzu HMV-G31ST Vickers microhardness tester (Shimadzu, Kyoto, Japan) following the Vickers method. Each test was averaged five times with a load of 2.942 N and a holding time of 10 s.

Finite Element Method (FEM) simulation was conducted using the Deform-3D software v. 13.1.1 (SFTC, Columbus, OH, USA). The material properties database and the initial model were taken from our previous work [50]. The only temperatures and reductions by rolling passes were changed. Finally, simulation conditions corresponded to the experimental.

## 3. Results and Discussion

**Figure 6:**
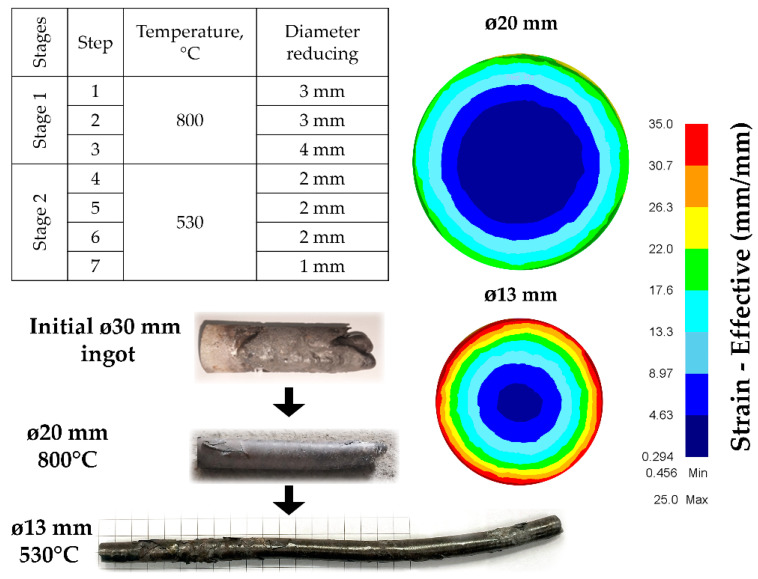
RSR-rolling scheme by stages and corresponding FEM-simulation outputs.

Figure 6 shows the progress of the experimental rolling of a zirconium ingot and a FEM model of its total cross-section deformations. Rolling the initial ingot at a high temperature (Stage 1) allows you to achieve a deformation level above 20 mm/mm on the surface and at least 3 mm/mm in the center of the sample. This should be enough to brew casting defects and transform the cast microstructure.

The second stage involves further rolling the resulting microstructure to a workpiece diameter of 13 mm at a reduced temperature to maximize the microstructure’s refinement. This increases the level of accumulated deformation to 35 mm/mm on the surface and significantly improves the study of the sample’s central regions.

**Figure 7:**
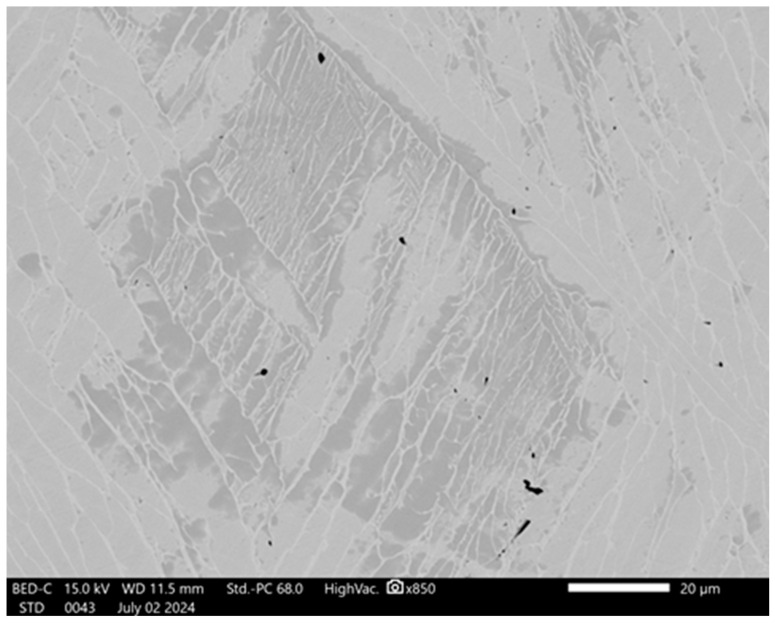
SEM (BED) is an image of the cast ingot structure.

After obtaining the simulation data, the change in the microstructure by rolling stages was studied to correlate them with the levels of deformation and their distribution. Figure 7 shows the microstructure of an ingot made of Zr-1%Nb alloy, smelted in an induction vacuum furnace, which shows the characteristic features of the cast structure. The grains have an elongated shape, which indicates the direction of crystallization due to the cooling conditions during vacuum melting.

There are also microdefects in the form of dark spots and lines in the image. Black spots are pores that have arisen during crystallization, which are due to the peculiarities of cooling and the possible influence of technological parameters of melting. Pores can significantly affect the mechanical properties of a material, contributing to a decrease in its density and deterioration of strength characteristics.

Thus, the microstructure of the molten ingot is characterized by dendritic structures, segregation of alloying elements, and the presence of porosity, which is the result of the casting processes and the specifics of induction vacuum melting. This is a typical acicular microstructure and is suitable for modeling ingot processing.

**Figure 8:**
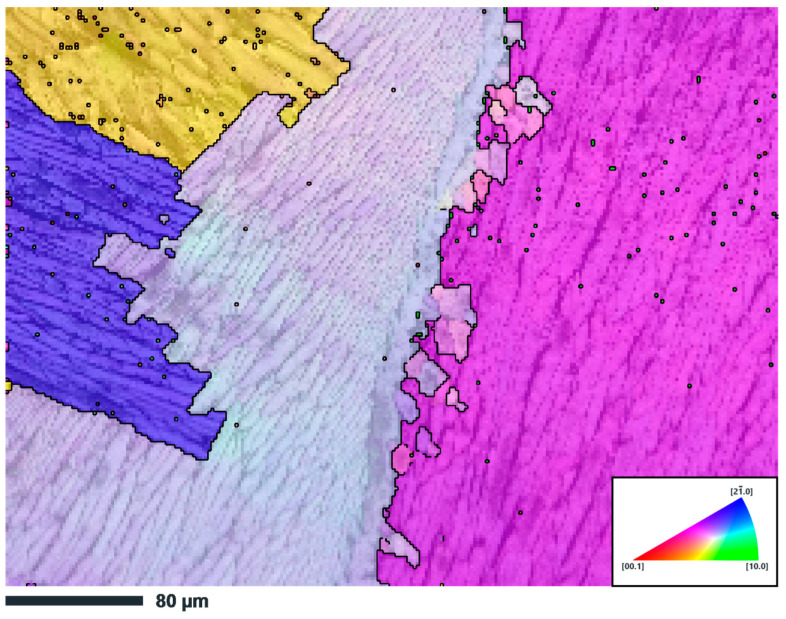
IPF map of the Zr-1%Nb alloy ingot sample.

Based on the conducted studies using the EBSD method of a zirconium ingot (Figure 8) smelted in an induction vacuum furnace, a complex crystallographic structure with pronounced heterogeneity was revealed. The grains have different crystallographic orientations, which are due to the uneven distribution of temperatures and heat fluxes during the solidification of the material. The grain boundaries are clearly distinguishable, which indicates the presence of significant textural differences in the cast structure characteristic of zirconium alloys cooled under vacuum conditions.

**Figure 9:**
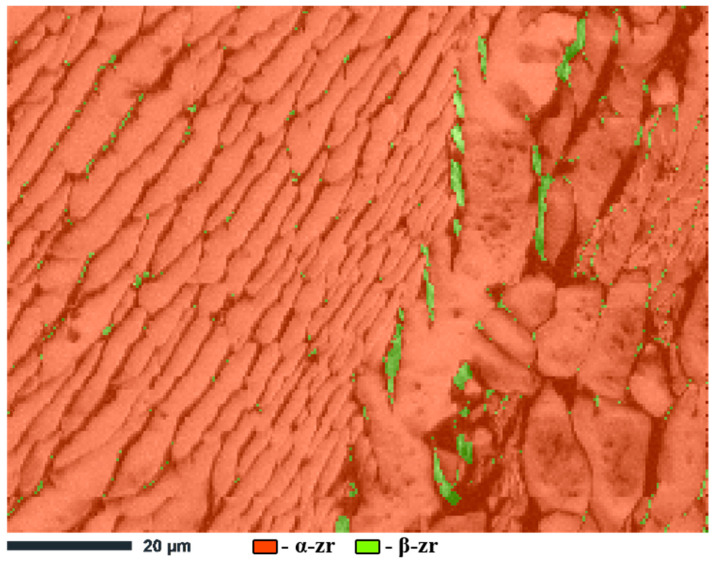
Phase EBSD mapping of a sample of a Zr-1%Nb alloy ingot.

Additionally, the phase map constructed using EBSD (Figure 9) confirms the presence of two phases in a zirconium ingot: the alpha phase of zirconium, which represents the main part of the microstructure, and the beta phase of zirconium, which is localized along grain boundaries. The alpha phase of zirconium, stabilized at room temperature, dominates the microstructure, having a hexagonal close-packed (HCP) crystal lattice. The beta phase of zirconium with a body-centered cubic (BCC) structure occurs as separate inclusions along the grain boundaries of the alpha phase, which indicates its residual character after solidification and incomplete transformation into the alpha phase due to rapid cooling.

Thus, the results of EBSD mapping and phase analysis indicate a complex microstructure of the Zr-1%Nb alloy ingot, characterized by both the heterogeneity of the crystallographic orientation of the grains and the phase composition. The presence of alpha and residual beta phases, along with the presence of large grains, underscores the critical importance of selecting appropriate thermomechanical processing techniques to enhance the material’s mechanical properties under operational conditions.

The results of the study of a zirconium ingot after radial-shear rolling at a temperature of 800 °C demonstrate significant changes in the microstructure along the entire radius of the bar. The sample was rolled from a diameter of 30 mm to 20 mm with local accumulated deformations of at least 3 mm/mm. For the analysis, measurements were performed using the EBSD method at five points along the radius of the rod—from the surface to its center every 2 mm.

**Figure 10:**
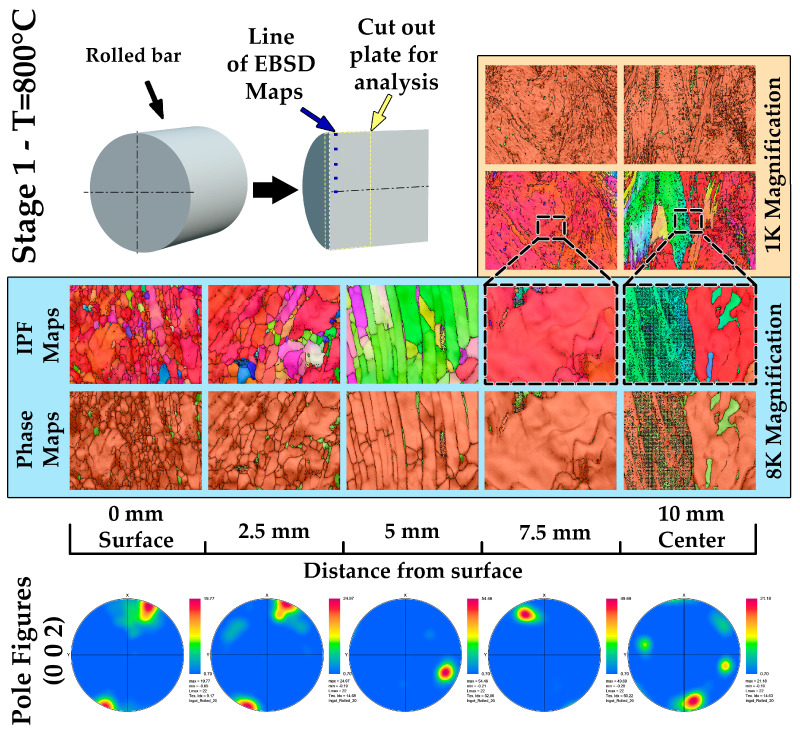
EBSD analysis of rolled zirconium ingot up to 20 mm.

The IPF maps in Figure 10 show that the most intense structural change occurs on the surface of the bar, which is associated with the peculiarities of plastic deformation during radial-shear rolling [41]. As a result of this process, the grains on the surface are actively crushed. As we approach the center of the rod, the intensity of deformation decreases, and areas with a less pronounced initial structure are observed. In the center of the bar, a cast structure or a region of a transitional structure is preserved, which indicates insufficient deformation in this zone associated with a decrease in the effect of deforming forces.

Phase maps (Figure 10) confirm the presence of the zirconium alpha phase as the main phase; however, residual beta phase sites are present along grain boundaries, mainly in peripheral areas where deformation was most intense. In the center of the sample, where the cast structure is preserved, there are signs of minimal processing of the phase composition, indicating the absence of an active phase transition in this zone.

Pole figures (Figure 10) demonstrate changes in the crystallographic texture along the radius of the rod. The texture is most pronounced on the surface of the sample, which is associated with the active redistribution of grains under the influence of deformation. Closer to the center, the texture becomes less pronounced, which confirms the preservation of a cast or transitional structure.

Thus, radial-shear rolling of a zirconium ingot at a temperature of 800 °C leads to intensive refinement of grains on the surface of the bar, while the central part retains a cast or partially deformed structure. These changes indicate a significant influence of the strain distribution over the cross-section of the bar and can significantly affect the mechanical properties of the material.

After radial-shear rolling of a zirconium ingot to a diameter of 13 mm at a temperature of 530 °C, a detailed assessment of changes in the microstructure and phase composition was carried out. Rolling was carried out with a gradual decrease in the diameter of the bar by 2 mm at each stage, with the exception of the last step, where the decrease was 1 mm. For the analysis, measurements were performed using the EBSD method at five points along the radius of the rod—from the surface to its center at the same distances every 1.625 mm.

**Figure 11:**
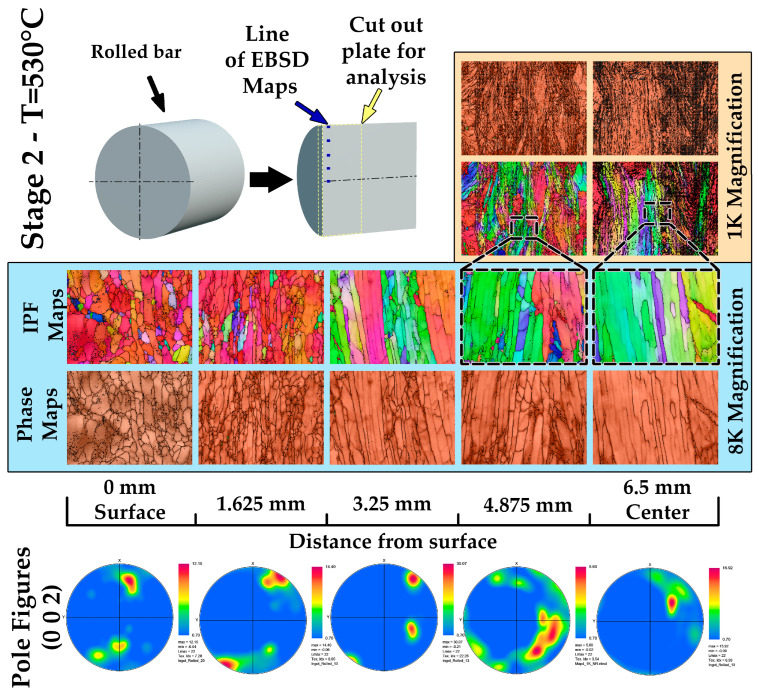
EBSD analysis of rolled zirconium ingot up to 13 mm.

Microstructural analysis (Figure 11) showed that significant grain crushing occurred at the periphery of the rod, and they reached submicron sizes. This is due to intense plastic deformation, which is most actively manifested on the surface of the rod. Dynamic recrystallization processes are likely to occur, which contributes to the formation of a fine-grained structure and improvement in material properties. As they approach the center of the bar, the grains acquire an elongated shape along the rolling direction, which indicates less active shear deformation in this area and the predominance of laminar metal flow. The central zone has retained pockets of large and elongated grains characteristic of areas with less intense deformation, but the overall structure remains crushed compared to the initial cast state.

The phase composition (Figure 10) of the rod has also undergone significant changes. The beta phase of zirconium, which was present in the early stages of processing, has almost completely disappeared. This is due to the fact that the temperature of 530 °C is insufficient to stabilize the beta phase, and as a result of cooling, a complete transition to the alpha phase occurred. The dominance of the alpha phase confirms that the selected rolling mode and temperature range contributed to the phase equilibrium in which the alpha phase of zirconium is the most stable under these conditions.

Pole figures (Figure 10) reflect changes in the crystallographic texture in various parts of the rod. On the surface of the rod, the field figures show a pronounced anisotropic texture, which is associated with a high intensity of deformation and grain redistribution. The grains are mainly oriented along the rolling direction. As they approach the center of the rod, the field figures show a decrease in anisotropy, which indicates a less pronounced deformation in this zone. However, even in the central part, the directionality of the texture remains, which confirms the active effect of radial-shear rolling on the entire volume of the ingot.

Thus, rolling up to 13 mm at a temperature of 530 °C led to a strong refinement of the grains on the surface of the rod, where their size reached a submicron level, and in the center, there is an elongated grain structure along the rolling direction. The phase composition after rolling is characterized by an almost complete absence of the beta phase, which is due to the transition to a stable alpha phase. These changes indicate an improvement in the properties and homogeneity of the material, which is important for the further manufacture of structural elements.

**Figure 12:**
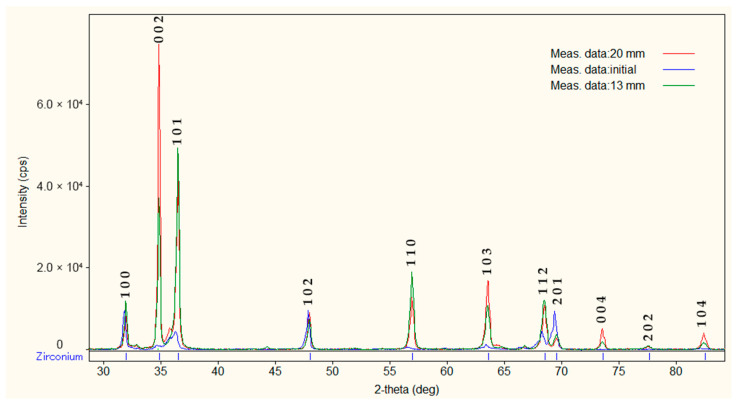
Evolution of the phase composition of a zirconium alloy based on the results of the XRD analysis.

X-ray diffraction analysis (XRD) was performed to analyze the phase composition. Figure 12 shows the results of X-ray phase analysis (XRD) of zirconium samples in the initial state, as well as after their processing to diameters of 20 mm and 13 mm by radial-shear rolling. The results of the XRD analysis showed the presence of the alpha phase of zirconium; peaks characteristic of the beta phase were not found. The absence of the beta phase on the XRD spectrum can be explained by its low content in the sample structure.

Analysis of the diffraction data revealed several intense peaks, the key of which is located at 34.61°, which corresponds to the plane (002) of the hexagonal close-packed (HCP) zirconium structure. This peak is the main feature of the α-phase of zirconium, which dominates the samples. In addition, other noticeable peaks at angles of 36.315° (plane (101)), 47.999°, and 56.955° also relate to the GPU structure, which further confirms the presence of the α-phase.

In the initial state and after rolling to different diameters, a similar pattern is observed with slight variations in peak intensity, which may be due to changes in texture or grain size caused by deformation during rolling. Nevertheless, the structure remains predominantly α-phase in all cases.

The presence of the β-phase of zirconium with a body-centered cubic (BCC) structure characteristic of the high-temperature state of zirconium is usually accompanied by peaks at angles of 38°, 55°, and 70°. However, on the presented radiograph, such peaks are weakly expressed or completely absent, which indicates a minimum or complete absence of the β-phase in the samples after processing.

The XRD results show that the beta phase of zirconium is almost completely absent after rolling, and the structure mainly consists of the alpha phase. This is an important observation, but the discussion on why the beta phase disappears and its influence on the material’s properties could be expanded. How does this phase change affect the operational characteristics of the alloy in nuclear applications?

During the heating and cooling of products made from the Zr-1%Nb alloy, phase transformations occur within the metal’s structure. The phase transition temperatures are as follows [49]: α-phase < 600 °C; 600 °C < α + β phase < 850 °C; β-phase > 850 °C. The presence and size of the β-phase precipitates along grain boundaries in finished products affect the results of corrosion tests. In traditional manufacturing processes of products from the Zr-1%Nb alloy, after hot deformation processing (in the temperature region where the α + β phase and β-phase exist), β-phase precipitates are usually found. Therefore, in subsequent multiple cycles of cold deformation processing, these precipitates need to be refined.

In this work, the authors selected optimal heating and radial-shear rolling regimes. The metal was heated to the temperature where only the α-phase exists. This allowed for the necessary workability of the metal for processing its structure with high shear deformations and minimized the conditions for phase transformations. Despite the short-term deformation heating of the metal to the temperature range where both α + β phases exist, it was important to establish that β-phase precipitates were not detected, which was confirmed by the results of XRD studies. Most likely, the accumulated energy resulting from intensive plastic deformation in the metal was sufficient so that in local areas (where the rolling temperatures due to deformation heating were in the α + β phase region), the phase transition α + β → α was completed.

Thus, the described manufacturing technology solves the problem of fragmenting the cast structure and eliminating β-phase precipitates in two cycles of radial-shear rolling, with a significant reduction in the traditional production cycle and metal losses in trimming.

A comparison of the diffraction data for the initial state and samples rolled to a diameter of 20 mm and 13 mm shows that changes in the phase composition are minimal; however, slight changes in peak intensity are observed, especially for the sample with the smallest diameter (13 mm). This indicates a change in the rolling process of the preferred orientations of grains.

The X-ray phase analysis showed that the structure of all samples after radial-shear rolling consists of the α-phase of zirconium, with an HCP crystal lattice, without signs of a significant amount of beta phase. Differences in peak intensity between the initial state and the samples after rolling indicate the effect of deformation on grain texture and size, which requires further research to better understand changes in microstructure SPD processing.

**Figure 13:**
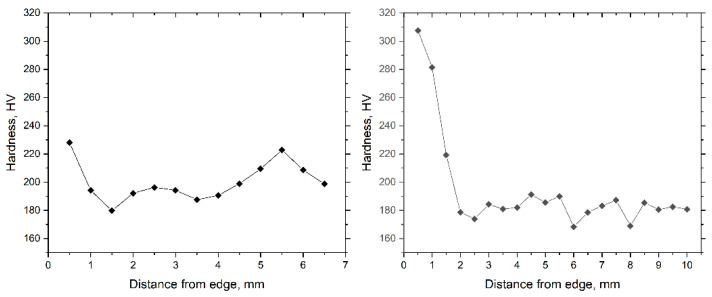
Microhardness distribution from the surface to the center in samples rolled up to 20 mm and 13 mm.

The presented graphs (Figure 13) show the results of measuring the microhardness of the ingot after rolling to diameters of 20 mm and 13 mm. Microhardness measurements were carried out for each rolled sample from the edge to the center in 0.5 mm increments, with five measurements at each point, and then the average value was calculated. The initial cast sample, before rolling, had a microhardness of 432 HV, which is typical for a coarse-grained cast structure and the presence of a residual zirconium beta phase.

The results for a sample rolled up to 20 mm show a sharp decrease in hardness from the edge to the center. On the surface of the bar, the hardness reaches maximum values of about 320 HV, which is explained by intense plastic deformation and significant grain refinement in the surface layers. However, as we move towards the center, the hardness gradually decreases, reaching minimum values of about 160–180 HV, which indicates the preservation of larger grains and a lower degree of deformation in the center of the bar.

A sample rolled up to 13 mm shows a similar trend, but with higher microhardness values compared to a sample rolled up to 20 mm. On the surface, the hardness is about 260 HV, which also indicates active grain refinement and accumulation of deformation in this area. In the central zones, there is a slight decrease in hardness, but the values remain higher than for a sample rolled up to 20 mm, which indicates a more uniform distribution of deformation and more intensive refinement of the structure.

Thus, the results show that rolling up to 13 mm at a higher strain intensity ensures a more uniform refinement of the structure over the entire bar cross-section, which is confirmed by increased microhardness values compared with a sample rolled up to 20 mm. This dependence of microhardness on the distance from the surface to the center of the sample indicates a significant influence of the strain intensity on the material’s mechanical properties.

The gradient of microhardness and microstructure fully correspond to the FEM-simulation data. The deformation level above 3 mm/mm in the center of the sample provides the achievement of the total transformation of the casted dendritic structure.

In general, it can be noted that the intense deformations inspired during ingot rolling by the RSR method make it possible to obtain a submicron structure in two stages, similar to that which can be obtained by other SPD methods previously noted in the introduction [30,31,32,33,34,37,38]. However, the resulting structure, unlike the structure obtained by the ECAP method, is not as uniform in the cross-section and has a gradient. However, unlike the blanks obtained by the ECAP method, the RSR rolling method used here makes it possible to obtain long blanks so demanded by industry. Unlike ARB, which is suitable only for obtaining flat sheets, the RSR method makes it possible to obtain blanks of round cross-sections suitable for the manufacture of components for nuclear power plants. As shown in [50], the RSR method, under better initial conditions, makes it possible to obtain better results, no worse than ECAP. The whole question is in optimizing the conditions. To achieve this, larger ingots should be used, allowing for greater total reductions.

## 4. Conclusions

(1) The application of the RSR method has demonstrated its effectiveness for rolling zirconium alloy ingots. The initial coarse-grained acicular microstructure of the ingot was significantly changed, which confirmed the possibility of successful processing of ingots using RSR. The ingot had a high microhardness (432 HV) due to the structure with acicular microstructure and the presence of a residual beta phase. However, rolling led to significant changes in the structure and phase composition, improving the mechanical properties of the material.
(2) Rolling up to a diameter of 20 mm at a temperature of 800 °C revealed an uneven distribution of deformation over the cross-section. The outer zone of the ingot demonstrated intensive grain refinement, while the central part retained a transitional structure close to the cast state. The partial conversion of the beta phase to the alpha phase confirmed the effectiveness of the RSR treatment for phase stabilization.
(3) Rolling up to 13 mm at a temperature of 530 °C turned out to be more efficient, which led to the refinement of grains to submicron sizes on the surface and the formation of an elongated grain structure in the center. The almost complete disappearance of the beta phase and the predominance of the alpha phase confirm the successful redistribution of deformation and phase stabilization under the selected rolling parameters.
(4) The microhardness of the samples decreased from the surface to the center, with maximum values in the peripheral zone subjected to the greatest deformation. Rolling up to 13 mm provided a more uniform distribution of hardness and grain crushing, which led to an improvement in the mechanical characteristics of the material.

Thus, radial-shear rolling has shown its applicability for processing cast ingots of zirconium alloy Zr-1%Nb. The RSR method made it possible to obtain rods with a submicron structure on the periphery and an elongated structure in the axial part. Rolling at a temperature of 530 °C and reducing the diameter to 13 mm demonstrated good results, which makes RSR a promising method for the production of fuel element structural parts from zirconium-based alloys.

Achieving an ultrafine-grained structure improves radiation tolerance and corrosion resistance due to the large number of grain boundaries within the volume of the part. Grain boundaries serve as drain surfaces for radiation defects and increase the length of the corrosion path deep into the part. The value of the work is in bringing the methods for obtaining such materials closer to industrial technologies for manufacturing components.

This study demonstrates the feasibility of using the RSR method to process ingots efficiently. Further work will need to focus on optimizing the RSR process parameters to achieve a more homogeneous microstructure across the entire cross-section and studying the mechanical behavior of the processed material under operating conditions typical of nuclear reactors, including assessing radiation resistance and corrosion behavior. In addition, it will be crucial to study the scalability range of RSR for industrial applications and its seamless integration into existing manufacturing processes. The ability to use severe plastic deformation to process long workpieces, achieve significant grain refinement, and improve their properties suggests that RSR can be integrated into existing production lines. A key feature for the potential industrial application of the method is its flexibility and efficiency, especially in demand for small-scale production of special materials.

## Data Availability

Data are contained within the article.
